# Successful Management of Refractory Type 1 Renal Tubular Acidosis with Amiloride

**DOI:** 10.1155/2017/8596169

**Published:** 2017-01-03

**Authors:** Patrick Oguejiofor, Robert Chow, Kenneth Yim, Bernard G. Jaar

**Affiliations:** ^1^Department of Internal Medicine, University of Maryland School of Medicine, Baltimore, MD, USA; ^2^Division of Nephrology, University of Maryland Medical Center Midtown Campus, Baltimore, MD, USA; ^3^Department of Medicine, Division of Nephrology, Johns Hopkins School of Medicine, Baltimore, MD, USA; ^4^Department of Epidemiology, Johns Hopkins Bloomberg School of Public Health, Baltimore, MD, USA; ^5^Nephrology Center of Maryland, Baltimore, MD, USA

## Abstract

A 28-year-old female with history of hypothyroidism, Sjögren's Syndrome, and Systemic Lupus Erythematosus (SLE) presented with complaints of severe generalized weakness, muscle pain, nausea, vomiting, and anorexia. Physical examination was unremarkable. Laboratory test showed hypokalemia at 1.6 mmol/l, nonanion metabolic acidosis with HCO_3_ of 11 mmol/l, random urine pH of 7.0, and urine anion gap of 8 mmol/l. CT scan of the abdomen revealed bilateral nephrocalcinosis. A diagnosis of type 1 RTA likely secondary to Sjögren's Syndrome was made. She was started on citric acid potassium citrate with escalating dosages to a maximum dose of 60 mEq daily and potassium chloride over 5 years without significant improvement in serum K^+^ and HCO_3_ levels. She had multiple emergency room visits for persistent muscle pain, generalized weakness, and cardiac arrhythmias. Citric acid potassium citrate was then replaced with sodium bicarbonate at 15.5 mEq every 6 hours which was continued for 2 years without significant improvement in her symptoms and electrolytes. Amiloride 5 mg daily was added to her regimen as a potassium sparing treatment with dramatic improvement in her symptoms and electrolyte levels (as shown in the figures). Amiloride was increased to 10 mg daily and potassium supplementation was discontinued without affecting her electrolytes. Her sodium bicarbonate was weaned to 7.7 mEq daily.

## 1. Introduction

Distal (type 1) renal tubular acidosis (RTA) is characterized by a decrease in net H^+^ secretion in the renal collecting tubule resulting in urinary pH > 5.5 [1, 2]. The normal anion gap metabolic acidosis of type 1 RTA is typically associated with episodes of hypokalemia and nephrocalcinosis [1, 2]. We herewith present a patient with persistent severe symptomatic hypokalemia secondary to type 1 RTA who was refractory to standard therapy but responded to the addition of amiloride to her treatment regimen.

## 2. Discussion

Distal RTA (type 1 RTA) is a rare renal disorder characterized by normal anion gap hyperchloremic metabolic acidosis [3]. In this condition, the alpha intercalated cells of the cortical collecting duct of the distal nephron fail to secrete acid into the urine [1–3]. This inability to secrete acid results in failure to acidify the urine to pH < 5.5 [1–7]. Because renal excretion is the primary means of eliminating acid from the body, there is consequently a tendency towards systemic academia [3]. This leads to the clinical features of type 1 RTA, which are listed as follows. 

*Clinical Features of Distal RTA*

Normal anion gap hyperchloremic metabolic acidosis.
Hypokalemia.
Nephrocalcinosis.
Nephrolithiasis (related to an inability to acidify urine).
Hypercalciuria and low urinary citrate.
Loss of calcium from bones.

Type 1 RTA is either inherited or acquired [1, 8]. Inherited type 1 RTA can be either autosomal-dominant or autosomal-recessive [1, 8]. Autosomal-recessive type 1 RTA often presents in infancy, while autosomal-dominant type 1 RTA may not present until adolescence or young adulthood [8–10]. Some patients with autosomal-recessive distal RTA have been associated with sensorineural hearing loss [11]. In the acquired form, type 1 RTA can be caused by drugs, autoimmune diseases, or infectious etiologies. Many of the commonly acquired forms are attributed to Sjögren's Syndrome, SLE, viral hepatitis, treatment with amphotericin B, toluene toxicity, and pyelonephritis [2, 8, 12, 13].

The clinical manifestations of type 1RTA are usually related to the disease type and severity and whether it is acquired or inherited [3]. Patients can also present without symptoms. Both inherited and acquired forms can be associated with hypokalemia, which might result in muscle soreness, flaccid paralysis, and cardiac conduction abnormalities [3]. Of note, the most common cause of death in type 1 RTA is hypokalemia-induced cardiac dysrhythmia [3, 14].

Treatment includes correction of hypokalemia and alkali replacement [2]. The hypokalemia should be corrected first, as alkali replacement can worsen the hypokalemia with dangerous consequences. Correcting hypokalemia improves musculoskeletal symptoms if present [15]. Early treatment also reduces the incidence of nephrocalcinosis, recurrence of renal stones and progression to chronic kidney disease [7, 16].

The diagnosis of distal RTA in our case was made on the basis of the patient's clinical presentation of profound muscle weakness, normal anion gap metabolic acidosis associated with hypokalemia, nephrocalcinosis, elevated urine pH, and positive urine anion gap. The history of Sjögren's Syndrome and SLE in our patient is also compelling, as distal tubular acidosis has been well documented to be associated with a variety of autoimmune disorders [1, 8].

The mechanism by which Sjögren's Syndrome leads to distal renal tubular acidosis is incompletely understood. In these patients, the absence of H^+^-ATPase pump in the intercalated cells of the collecting tubules has been documented, which can result in the inability to secrete protons and acidify urine [2].

**Figure 1:**
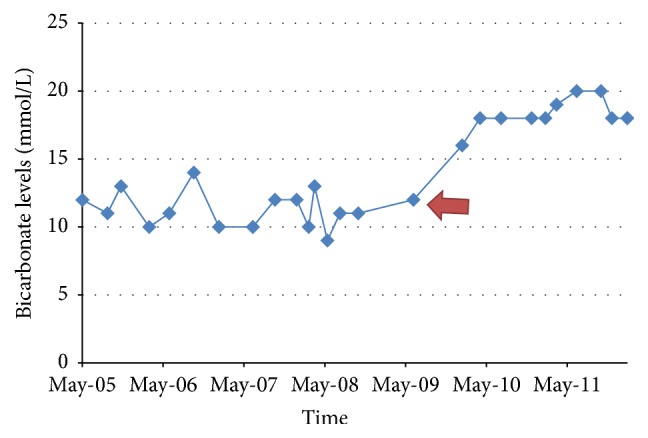
Bicarbonate levels over time. Red arrow indicates initiation of amiloride.

**Figure 2:**
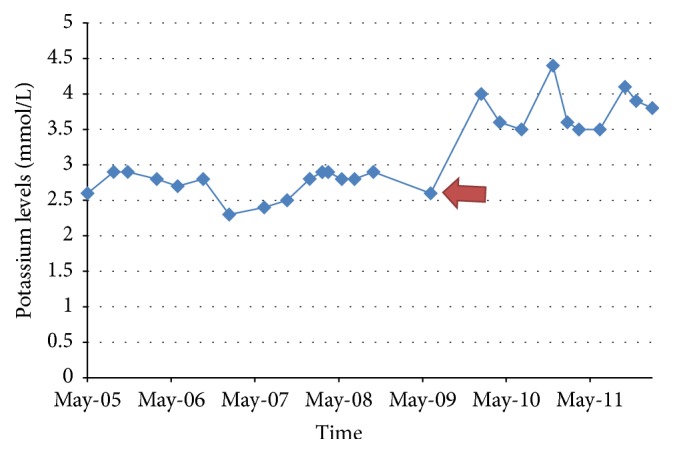
Potassium levels over time. Red arrow indicates initiation of amiloride.

In our case, the patient was resistant to the usual treatment of type 1 RTA but finally responded to a trial of amiloride, with improvement in both serum K^+^ and HCO_3_ levels (see Figures 1 and 2). No other interventions were undertaken at the time that would account for the improvement in hypokalemia and metabolic acidosis. She was adherent to her medication regimen as evidenced by regular medication refill as well as her compliance with her medical appointments.

Amiloride is potassium-sparing diuretic, inhibiting Na^+^ reabsorption at the distal convoluted tubule, cortical collecting tubule, and collecting duct. This action leads to decrease in potassium secretion and increase in serum potassium retention. The patient's symptoms and potassium level improved significantly and immediately upon addition of this medication. Her serum K^+^ level remained greater than 3.5 mmol/L after initiation of amiloride. In addition, her serum HCO_3_ level also somewhat improved over a 12-month period and remained stable, despite decreasing her daily dose of NaHCO_3_ from 15.5 mEq every 6 hours to her present dose of 7.7 mEq per day. The mechanism of the moderate improvement in our patient's serum HCO_3_ remains unclear at this point. It may be possible that she also had a component of proximal RTA that resolved over time; however, experimental studies have shown that chronic use of amiloride does not necessarily lead to negative values in net acid excretion over time thereby contributing less to maintenance of metabolic acidosis [17].

## 3. Conclusion

In retrospect, this case highlights some important learning points. (i) Musculoskeletal symptoms are a common clinical manifestation of RTA. (ii) Although not previously reported, amiloride can be used in the treatment of severe refractory symptomatic hypokalemia associated with type 1 RTA. (iii) Although uncommon, RTA should be included in the differential diagnoses in patients presenting with musculoskeletal symptoms and nephrocalcinosis.
